# Impact of Artificial Aging on the Physical and Mechanical Characteristics of Denture Base Materials Fabricated via 3D Printing

**DOI:** 10.1155/2024/8060363

**Published:** 2024-06-18

**Authors:** Ahmed Altarazi, Julfikar Haider, Abdulaziz Alhotan, Nick Silikas, Hugh Devlin

**Affiliations:** ^1^Division of Dentistry, Faculty of Biology, Medicine and Health, The University of Manchester, Manchester M13 9PL, UK; ^2^Restorative Dental Science, College of Dentistry, Taibah University, Medinah, Saudi Arabia; ^3^Department of Engineering, Manchester Metropolitan University, Manchester M1 5GD, UK; ^4^Department of Dental Health, College of Applied Medical Sciences, King Saud University, Riyadh, Saudi Arabia; ^5^School of Dentistry, University of Jordan, Amman, Jordan

## Abstract

Three-dimensional (3D) printing is becoming more prevalent in the dental sector due to its potential to save time for dental practitioners, streamline fabrication processes, enhance precision and consistency in fabricating prosthetic models, and offer cost-effective solutions. However, the effect of aging in artificial saliva of this type of material has not been explored. To assess the physical and mechanical properties of the two types of 3D-printed materials before and after being subjected to artificial saliva, a total of 219 acrylic resin specimens were produced. These specimens were made with two types of 3D-printed materials, namely, NextDent (ND) and Formlabs (FLs), and a Schottlander heat-cured (HC) resin material that was used as a control. Water sorption and solubility specimens (*n* = 5) were tested after three months of storage in artificial saliva. Moreover, the Vickers hardness, Martens hardness, flexural strength/modulus, and impact strength were evaluated both under dry conditions and after three months of storage in artificial saliva. The degree of conversion (DC), elemental analysis, and filler content were also investigated. The ANOVA showed that 3D-printed resins had significantly greater sorption than the control group (*p* < 0.05). However, the flexural strength values of the 3D-printed materials were significantly greater (*p* < 0.05) than those of the heat-cured material. The DC of the 3D-printed resins was lower than that of the control group, but the difference was not significant (*p* > 0.05). The 3D-printed materials contained significantly more filler than the control (*p* < 0.05). Moreover, the artificial saliva had a significant effect on the Vickers hardness for all tested groups and on the Martens hardness for the control group only (*p* < 0.05). Compared with conventional heat-cured materials, 3D-printed denture base materials demonstrated relatively poorer performance in terms of sorption, solubility, and DC but exhibited either comparable or superior mechanical properties. The aging process also influenced the Vickers and Martens' hardness. The strength of the 3D-printed materials was in compliance with ISO recommendations, and the materials could be used alongside conventional heat-cured materials.

## 1. Introduction

Significant efforts have been made to improve the quality of polymethyl methacrylate (PMMA) materials and thereby overcome the known disadvantages associated with the traditional method of denture manufacturing [1]. The production of digital denture templates has been made possible due to recent advancements in computer-aided design and computer-aided manufacturing (CAD/CAM) technology and its associated software [2, 3]. A Standard Tessellation Language (STL) file is created once the denture has been digitally designed, and then either a subtractive (computerized numerically controlled milling) or an additive (3D printing) method can be used to produce the denture [4, 5].

Several benefits of CAD/CAM-fabricated prostheses include fewer appointments, streamlined denture manufacturing processes, greater tissue adaptability, simple replication of preexisting dentures, fewer manufacturing errors, and more rapid production [3, 6–8]. Although milling is a common method for making dentures, 3D printing offers important benefits. For instance, it is more cost-effective since it allows for the simultaneous production of several components without wasting raw materials or wearing rotary tools [9, 10].

The mechanical properties of any polymer for oral use, regardless of its manufacturing technology, could be compromised by oral cavity fluids due to the absorption of water by acrylic, which could compromise the physical properties of the polymer [11]. Denture bases manufactured via 3D printing must adhere to strict standards for oral stability to guarantee prolonged use. These standards encompass minimal water absorption and solubility, elevated mechanical properties to withstand chewing forces, and a substantial degree of conversion to mitigate biological impacts from residual monomers [12, 13]. The interplay between water and polymer chains can induce internal strains through swelling, chemical breakdown, and residual monomer release, underscoring the critical importance of sorption and solubility as key metrics for evaluating the durability of denture resins [14, 15]. In addition, maintaining the lowest possible amount of unreacted monomers is essential for ensuring excellent biocompatibility. The degree of conversion (DC) of the resin can be measured to predict the amount of unpolymerized monomer that can irritate and harm a patient's oral mucosa [16].

On the other hand, monitoring variations in hardness after preserving specimens in solvents can be used to indirectly examine polymer degradation. The hardness of a solid material can be measured by assessing its level of resistance to a compressive force applied to its surface by an indenter [17, 18]. Studies of this nature typically assess the hardness after the load has been removed. Although this approach is useful for materials that are flexible plastically, elastic-plastic materials are less accurately described in this way [18]. The Martens hardness method was devised to address these constraints. This technique integrates sensors capable of recording elastic and plastic deformation components during force- or depth-controlled instrumented indentation, employing a conventional Vickers diamond tip for loading. The indentation modulus, creep, and depth are among the numerical parameters automatically extracted from the resultant force-displacement plots at each instance. Although several studies have assessed Martens hardness for different polymer-based composites and ceramics [19–21], there is a need to study the surface stability of 3D-printed denture base polymers.

There are few investigations comparing the physical and mechanical characteristics of 3D-printed and aged denture base acrylic resins that are produced hydrolytically using artificial saliva. In the authors' previous study, a single type of 3D-printed resin with various concentrations of TiO_2_ nanofillers was tested, and the physical and mechanical properties were assessed to determine the effect of the filler particles [22]. In the current research, two types of plain 3D-printed materials and conventional heat-cured materials were studied, allowing for a comprehensive comparison of their physical and mechanical properties. In this study, the flexural strength, flexural modulus, impact strength, surface hardness, including Martens hardness (HM) and Vickers hardness (HV), sorption and solubility, and degree of polymerization of 3D-printed denture base resins were compared with those of traditional heat-polymerized acrylic resin both before and after hydrolytic aging in artificial saliva. The first null hypothesis was that these physical and mechanical properties of the 3D-printed resins would not differ significantly from those of traditional heat-polymerized resins either before or after artificial aging.

## 2. Materials and Methods

### 2.1. Resin Material

The first 3D-printed material examined in this investigation was a commercially available NextDent (ND) 3D + light-cured resin with a light pink hue (3D Systems, Soesterberg, Netherlands) that was specifically engineered for denture base applications. According to the manufacturer's specifications, NextDent possesses the following characteristics: an ultimate flexural strength of 84 MPa, a flexural modulus of 2383 MPa, a sorption rate of 28 g/mm^3^, and a solubility of 0.1 g/mm^3^. The second 3D-printed material utilized in this research was a Formlabs (FLs) digital denture resin, which was also light pink (Formlabs, Somerville, USA). Although the manufacturer did not disclose the exact properties of the material due to proprietary concerns, the resin composition is detailed in the safety data sheet, as provided in Table 1. For comparative analysis, heat-cured (HC) PMMA served as the control material. To produce the specimens, a combination of powder and liquid monomers of methyl methacrylate (Pegasus Plus, Schottlander, Hertfordshire, UK) was used.

### 2.2. Sample Fabrication and Aging Procedure

**Figure 1:**
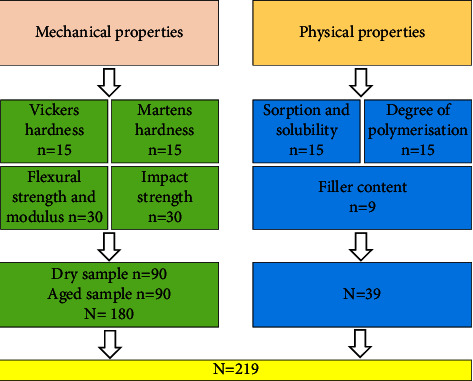
Comprehensive plan for 3D-printed sample characterization.

Fabrication and aging processes were carried out on a total of 219 specimens as shown in Figure 1 (*n* = 73 per material), following established protocols as described in a prior study [23]. In brief, the Formlabs Form 2 printer was employed, with PreForm software facilitating CAD design editing and vertical positioning (90°). The specimens were cleaned in 99.9% isopropyl alcohol (IPA) or 99.8% ethanol for ND, followed by air drying. Subsequently, they were immersed in preheated glycerine at 80°C and cured at the same temperature for 30 minutes using a light chamber box (Form Cure, Formlabs, Somerville, USA), with the ND specimens subjected to additional polymerization in a UV light box at 60°C.

All specimens underwent wet grinding to achieve flat, smooth edges and faces using silicon carbide grinding papers of varying grain sizes (approximately 30 *μ*m, 18 *μ*m, and 15 *μ*m). A digital caliper with an accuracy of 0.01 mm was used to measure the specimen dimensions. HC material was manufactured following an established protocol detailed in a previous study [22].

The specimens were evaluated for their Vickers hardness (HV), Martens hardness (HM), flexural strength, and impact strength after being placed in artificial saliva and kept at 37°C for three months [22]. The solution was replenished every 14 days.

### 2.3. Analysis of Filler Content

The percentage of inorganic components in each type of resin material used in this study was measured by eliminating the organic component of the resin materials. Following the ISO standard 1172: 2022 (BS EN ISO 1172:2022), three specimens from a resin material were subjected to heating in an electric furnace (Programat EP 5000; Ivoclar Vivadent, Liechtenstein, Austria) within a temperature range of 470°C–500°C for 15 minutes, followed by cooling in a desiccator. Subsequently, each specimen's weight was measured using a precision electronic balance (Ohaus Analytical Plus, Ohaus, USA; accuracy: 0.01 mg). The percentage of inorganic fillers was calculated by using the following equation:

(1) $$\begin{array}{c} \text{Filler weight }\%=\frac{m_{3}-m_{1}}{m_{2}-m_{1}}\times 100, \end{array}$$

where *m*_1_ indicates the initial mass of the empty crucible in grams and *m*_2_ is the initial mass of the crucible along with the dried specimen in grams. Finally, *m*_3_ denotes the end mass of the crucible, including the remaining mass of the specimen after it has been calcined, in grams.

### 2.4. Procedure for Determining Degree of Conversion (DC)

Fourier transform infrared (FTIR) spectroscopy was conducted to determine the DC of the specimens using a Spotlight 200i FT-IR microscope fitted with a Spectrum Two instrument at wavelengths between 4000 and 400 cm^−1^. Five specimens (diameter 15.0 ± 0.2 mm and thickness 2.0 ± 0.2 mm) of each resin were manufactured. DC was determined as the ratio of double carbon bond peaks at an aliphatic stretch frequency of 1637 cm^−1^ to the reference aromatic frequency of 1608 cm^−1^ by the following equation:

(2) $$\begin{array}{c} \text{DC}\left(\%\right)=\left(1-\left(\frac{\left(1637^{-1}/1608^{-1}\right)\text{ peak }he\text{ights after polymerisation}}{\left(1637^{-1}/1608^{-1}\right)\text{peak }he\text{ights before polymerisation}}\right)\times 100\right.. \end{array}$$

### 2.5. Evaluating Sorption Characteristics

According to the ISO 20795-1: 2013 standard, saliva sorption characteristics were determined for the specimens from each material (*n* = 5) with sample dimensions of 50.0 ± 0.5 mm in diameter and 1.0 ± 0.2 mm in thickness. The specimens were kept in a desiccator containing silica gel for 24 hours at 37°C and subsequently weighed using an analytical balance (Ohaus Analytical Plus, Ohaus, USA; precision: 0.01 mg) until a constant mass (*m*_1_) was obtained. The specimens were submerged in artificial saliva at a temperature of 37 ± 2°C. Their mass was measured after each withdrawal from the solution and subsequent drying until the difference between consecutive weighings was not more than 0.2 mg (*m*_2_). To obtain *m*_3_, specimens were reconditioned in a desiccator using the same method until they reached a stable mass. Equations (4)–(6) are used to calculate the sorption (g/mm^3^), solubility (g/mm^3^), and mass change (%), respectively.

(3) $$\begin{array}{c} \text{Specimen volume},V=3.14\times \left({\left(\frac{\text{mean diameter}}{2}\right)}^{2}\right)\times \text{mean thickness}, \end{array}$$

(4) $$\begin{array}{c} \text{Sorption}=\frac{m2-m3}{V}, \end{array}$$

(5) $$\begin{array}{c} \text{Solubility}=\frac{m1-m3}{V}, \end{array}$$

(6) $$\begin{array}{c} \text{Change in mass},\text{SP }\left(\%\right)=\left(\frac{m^{t}-\;m1}{m1}\right)\times 100, \end{array}$$

where “*m*^t^” denotes the mass of the specimen at a specific point in time.

### 2.6. Mechanical Properties Measurement

The mechanical properties of each material were evaluated, and the details are given in Table 2. Further details can be found in the authors' previous publication [22].

### 2.7. Optical Microscopy and Scanning Electron Microscopy (SEM)

The surface morphology of the polished printed specimens was assessed using an optical microscope (Echo Revolve, California, USA; magnification ×10). Furthermore, analysis of the fractured surfaces coated with a thin gold layer resulting from the flexural strength test was conducted using a scanning electron microscope equipped with an energy-dispersive X-ray spectrometer (SEM-EDX, Carl Zeiss Ltd., Cambridge, UK).

### 2.8. Data Analysis

The data were analyzed using SPSS version 25 (IBM, New York, NY, USA), and statistical tests were performed to ensure the accuracy of the findings. The normality of the data was assessed by the Shapiro‒Wilk test, while the homogeneity of the data was confirmed by the Levene test. One-way and two-way ANOVA tests were carried out to explore the interaction between the study materials and the storage media. Subsequently, Tukey's/Games–Howell post hoc analysis (*p* ≤ 0.05) was performed to delve deeper into any significant discrepancies.

## 3. Results

### 3.1. Filler Content

Table 3 represents the amount of filler measured in this study in comparison to the data provided by the manufacturers where available. The 3D-printed materials had significantly greater filler content than the HC material (*p* < 0.001). No significant difference was detected between ND and FL (*p* > 0.05).

EDX analysis was used to identify the elemental composition of the fillers within the 3D-printed resins, as the HC material was almost 100% PMMA. The results revealed the presence of aluminum (Al) within FL and a very minor trace of copper (Cu). EDX analysis also revealed the presence of aluminum (Al), silica (Si), titanium (Ti), and iron (Fe) within the ND resin material as nonorganic fillers. The ranges of inorganic fillers detected were 1–13 wt.% and 1–20 wt.% for FL and ND, respectively.

### 3.2. Degree of Conversion

**Figure 2:**
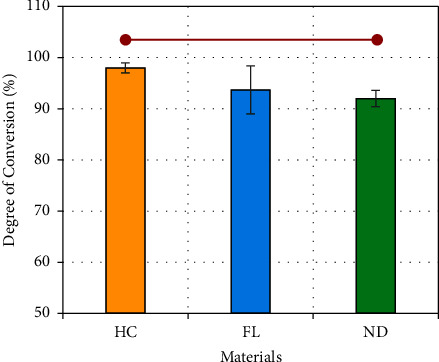
Degree of conversion (DC) of the three tested materials. A statistically significant difference is indicated by horizontal red lines connecting two points (Games–Howell post hoc test: *α* = 0.05 and *n* = 5).

Figure 2 presents the mean DC and standard deviation values of the resin materials. The HC group exhibited the highest DC among the tested materials, with a value of 97.2%. One-way ANOVA indicated that the mean values of the groups were significantly different (*p* < 0.02; *F* = 5.6). In addition, the Games–Howell post hoc comparison revealed that there was a significant difference between the HC and ND groups (*p* < 0.02). Compared with ND, FL also had a slightly greater percentage, but the difference was not significant (93.7% and 92.0%, respectively).

### 3.3. Sorption and Solubility Analysis

**Figure 3:**
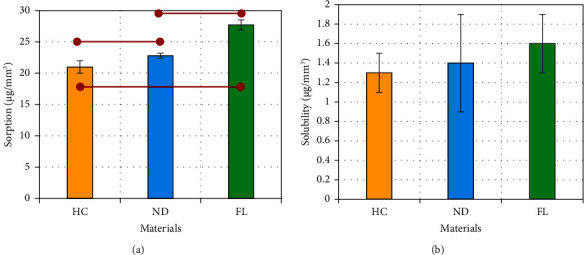
(a) Sorption and (b) solubility of the three tested materials observed for five weeks in artificial saliva. A statistically significant difference is indicated by horizontal red lines connecting two points (Tukey's post hoc test: *α* = 0.05 and *n* = 5).

**Figure 4:**
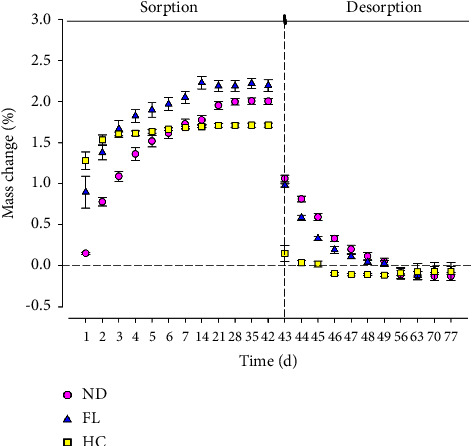
Sorption and desorption characteristics in the form of mass change when immersed in artificial saliva over 77 days.

Figure 3 displays the mean sorption values and standard deviations. Statistical analysis using one-way ANOVA revealed that there was a significant difference between the mean values of the groups (*p* < 0.001; *F* = 103.2). Tukey's post hoc analysis revealed that there was a significant difference between the HC group and the other 3D-printed groups (*p* < 0.001) and between the ND and FL groups (*p* < 0.001). However, one-way ANOVA did not reveal any significant difference between the mean values of solubility (*p* > 0.05). During the sorption process, HC showed a notable increase in mass within the initial 3 days. Following this, the mass increase plateaued, becoming negligible until day 42. This plateau indicates that the equilibrium has been reached, as shown in Figure 4. Similarly, during the desorption process, the change in mass of the specimens was mostly observed within the first 3 days, with negligible changes thereafter. In the case of the 3D-printed groups (ND and FL), different patterns were observed. The mass change occurred consistently for up to 21 days during the sorption process, with the increase becoming insignificant after this point and remaining so until day 42. In the desorption phase, the mass change was concentrated in the first 7 days, with insignificant variations in mass detected thereafter.

### 3.4. Surface Hardness

**Figure 5:**
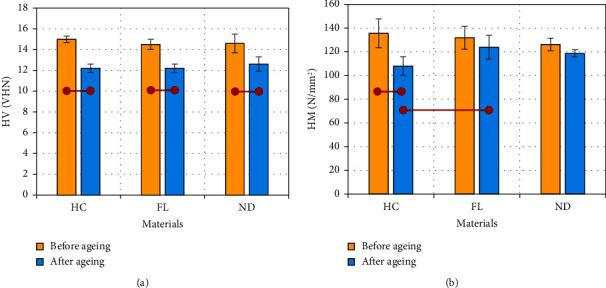
(a) Vickers and (b) Marten hardness for the three tested materials both before and after exposure to artificial saliva for three months. A statistically significant difference is indicated by horizontal red lines connecting two points (Tukey's post hoc test: *α* = 0.05 and *n* = 5).

The means and standard deviations of HV are presented in Figure 5(a). No significant difference was observed across different materials when comparing baseline readings (*p* > 0.05) or after the aging process. For the same material before and after aging, the values of HV decreased significantly in all groups (*p* < 0.001).  Figure 5(b) shows the mean and standard deviation of HM for different materials before and after aging. At baseline, no statistically significant differences were reported among the groups (*p* > 0.05). After the aging period, the HC group showed a significantly lower value than the FL resin group (*p* < 0.01; *F* = 6.5). However, no significant difference was reported between 3D-printed resin materials (*p* > 0.05). Within the same material, no significant difference was observed before and after the aging process for the 3D-printed resins, but the value for the HC group decreased significantly (*p* < 0.001).

### 3.5. Flexural Strength and Modulus

**Figure 6:**
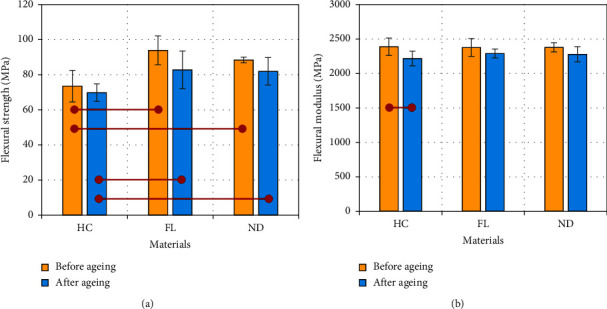
(a) Flexural strength and (b) modulus of the three tested materials before and after three months of aging in artificial saliva. A statistically significant difference is indicated by horizontal red lines connecting two points (Tukey's post hoc test: *α* = 0.05 and *n* = 5).

Figure 6 shows the means and standard deviations of the flexural strength and flexural modulus. The results show a significant increase in the flexural strength associated with 3D-printed resins compared to that of the HC material both before aging (*p* < 0.02; *F* = 39.5) and after aging (*p* < 0.03) across different materials. Following a 3-month aging process in artificial saliva, the values for all materials decreased, but the decrease was not significant (*p* > 0.05). The analysis of the flexural modulus revealed no significant difference between the tested groups before aging (*p* > 0.05). However, the aging process had a significant effect on the HC resin (*p* < 0.002).

### 3.6. Impact Strength

**Figure 7:**
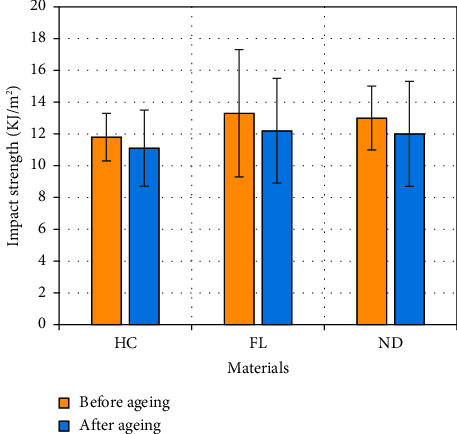
Impact strength of the tested materials before and after aging in artificial saliva for three months (Tukey's post hoc test: *α* = 0.05 and *n* = 5).

Figure 7 shows the mean values for the impact strength test. One-way ANOVA did not show any significant difference between all groups across the materials, and the *t*-test indicated no significant difference before or after the aging process within the same material (*p* > 0.05).

## 4. Discussion

In this study, the mechanical and physical properties of denture base materials manufactured using different technologies were investigated, with a focus on 3D printing technology. Both null hypotheses were partially rejected, as the statistical analysis revealed that 3D-printed specimens had significantly higher values in some tests than did the conventional heat-cured resin. In addition, the aging process significantly affects the materials in some tests.

In this study, sorption and solubility were assessed in artificial saliva to mimic the oral environment. The process continued until all tested groups reached full equilibrium, where the specimens could gain no more weight. This process lasted between 2 and 3 weeks for sorption and 1-2 weeks for desorption (solubility). According to the results of this study, the sorption of 3D-printed denture base materials was greater than that of the control heat-cured material, and this finding is in agreement with other studies [14, 15, 24]. This increased sorption associated with 3D-printed materials can be attributed to several factors, including the degree of conversion [15, 23] and the chemical composition of the materials [15, 25, 26].

Based on the results of this study, a negative correlation was found between DC and the sorption/solubility performance. The DC of the tested materials in this study revealed that the HC material had a higher DC than its 3D-printed counterparts. A higher DC generally results in a more densely cross-linked polymeric network that leaves less unreacted monomers and less space for water molecules within the material [12, 27–29]. As a result, materials with higher DC typically have lower sorption rates. One of the possible reasons for the increased DC of the HC material compared to that of the 3D-printed materials is the manufacturing technology. The HC material is polymerized under a higher temperature and pressure and for a longer time (cycling for six hours in a water bath); this could have a positive effect on the DC [30–32]. Another possible reason is the filler content of the material. To calculate the amount of filler in each type of material, the ash technique was performed in addition to EDX analysis. FL and ND had significantly greater amounts of inorganic fillers than HC. This finding established a relationship between filler content and DC, as more inorganic fillers within the material resulted in lower DC. Although the relationship between the two is complex, the presence of more inorganic components can hinder the DC in some ways: (1) a high filler content may interfere with the polymerization reaction by physically hindering the movement of reactive sites, making it more difficult for monomers to come into close contact and react with each other. This can result in a lower DC and (2) certain types of inorganic fillers can scatter or absorb light, which may reduce the available energy to initiate the polymerization process [33–35]. In general, the relationship between the inorganic filler content and DC is not directly straightforward, as the filler content, morphology, distribution, quality of the filler-matrix interface, and matrix composition must be considered.

Another aspect to look at is the interlayering spaces found within FL and ND due to their manufacturing technology. Gad et al. [14] and Greil et al. [24] reported similar results to those of this study in regard to water sorption, as 3D-printed denture materials absorbed more water than conventional materials. They explained that this was due to the presence of voids and interlayer spaces in 3D-printed specimens due to their manufacturing process, which can accommodate water molecules. However, this phenomenon was not observed in our previously published study [36], as optical microscopy and SEM analysis confirmed the absence of any interlayering spaces.

The solubility of the material is defined as the amount of components, including water-soluble elements, plasticizers, initiators, and unreacted monomers, that leach out of the specimen when immersed in water (or any other solvent) [37]. Many studies have shown that the solubility of 3D-printed materials is greater than that of the heat-cured materials or pressed resin materials [14, 15, 24, 38]. In this study, a similar observation was found, as the 3D-printed materials showed higher solubility than the control in the following order: FL > ND > HC. However, the difference between all the tested materials was insignificant. Notably, all tested materials were compliant with the ISO 20795-1-2013 recommendations in terms of sorption and solubility (32 *μ*g/mm^3^ and 1.6 *μ*g/mm^3^, respectively).

3D-printed resins are photopolymerized resin materials, and the DC plays a crucial role in determining the overall performance of the denture base. DC can influence a material's water absorption behavior, as a densely cross-linked network is less susceptible to water penetration, as previously explained. It is also important to consider the chemical composition of the material along with the DC, as DC can also play an important role in the water absorption capacity and durability of the resin material.

HV represents the ability of the denture base to resist abrasion, scratches, and indentation on the surface during function, especially when chewing hard substances or cleaning the denture with a toothbrush after use to prevent plaque accumulation and pigmentation [39, 40]. In this study, similar patterns were observed between 3D-printed specimens and the HC group at baseline. The HV of the HC specimens was slightly higher but not significantly different from that of the 3D-printed specimens. Like our results, other studies in the literature reported the same observations [12, 41].

The HM parameters used in this study were adopted from another study [20], which was suitable for use with polymer-based materials. At baseline, the HM results were similar to those of the HVs, as the HC specimens were slightly higher than those in the 3D-printed groups, but no significant difference was observed. The HM results in this study were in agreement with those of other studies that reported a range of 116–183 N/mm^2^ for heat-cured denture base materials [42] and 109–142 N/mm^2^ for 3D-printed PMMA-based materials [43].

**Figure 8:**
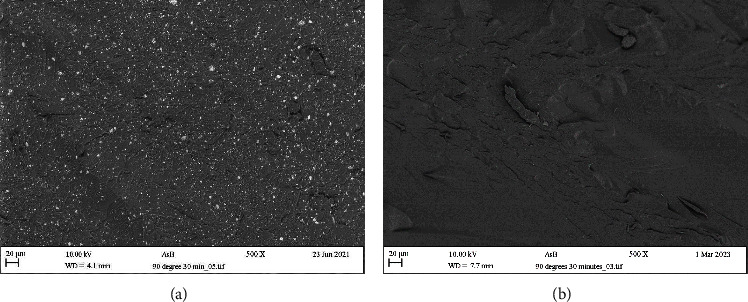
SEM images of the cross-sectional surface morphology of (a) NextDent and (b) Formlab.

Flexural strength is a critical property for denture base materials because it reflects their ability to withstand bending and twisting forces in the oral environment generated during normal functioning and natural movements. Conclusions regarding the comparison of flexural strength values between 3D-printed and conventional heat-cured materials vary in the literature. Some studies reported that 3D-printed materials showed lower values than heat-cured materials [23, 41]. Other studies reported comparable results [12, 44]. In contrast, some studies reported that 3D-printed materials showed higher values than conventional materials [24]. This variation in the comparison may be related to the settings used during the printing of the 3D-printed specimens and the composition of the materials used in the comparison. In 3D printing manufacturing, photopolymerized resin materials are used, and these materials depend on printing parameters and postcuring procedures [45–47]. In this study, the printing orientation and postcuring process followed another study [36], where a vertical orientation (90° to the build platform) and 30-minute curing time settings were used to obtain the optimal mechanical and physical properties for the same material. Most studies do not report the printing orientation used; however, Perea-Lowery et al. [15] reported using a horizontal orientation and indicated a lower flexural strength of the 3D-printed material compared to the conventional material. On the other hand, Greil et al. [24] used a vertical-printing orientation and claimed that 3D-printed specimens produced had higher flexural strength values than conventional materials. This is in line with previous study's findings [36], as the vertical orientation produces better mechanical properties than the horizontal printing orientation. Furthermore, some studies have reported that the weaknesses associated with 3D-printed materials may be related to the layering structure within the specimens, potentially leading to poor mechanical properties due to internal defects [23]. However, this study's SEM analysis of fractured surfaces did not reveal such defects (Figures 8(a) and 8(b)), which aligns with the findings of other researchers [36, 48, 49]. These findings could explain the variety in the literature regarding different conclusions about mechanical properties. In this study, compared with those in the conventional HC group, the flexural strength and modulus of ND and FL in the vertical-printing group were greater. One aspect to consider when explaining the differences between heat-cured resin and 3D-printed resin materials is the filler content. Polymeric materials primarily consist of a polymer matrix (organic component) and reinforcing fillers (inorganic component) [50, 51]. The amount of inorganic components directly affects the mechanical properties of the material [50]. In this study, it was discovered that the amounts of filler particles in ND and FL were greater than those in HC, which could further explain the superior mechanical properties associated with 3D-printed materials. Another aspect of utmost importance is the chemical composition and structure of the resin materials. The main composition of the HC material used in this study was PMMA, while dimethacrylate-based polymers were used for FL and ND. Dimethacrylate and PMMA are both acrylic-based polymers, but they have some differences in their chemical structure and properties [52, 53]. Dimethacrylates are compounds with two methacrylate groups, often connected by a spacer molecule. They are typically formed by the reaction of methacrylic acid with a diol or other difunctional molecule. PMMA is a polymer derived from the polymerization of methyl methacrylate (MMA) monomers. The chemical structure consists of repeated units of methyl methacrylate linked together to form a linear chain. PMMA can form cross-linked structures, but the degree of cross-linking is generally lower than that of dimethacrylate-based resins. In contrast, dimethacrylate-based resins are specifically designed to create highly cross-linked polymer networks. This is because the presence of two methacrylate groups allows for the formation of multiple covalent bonds between polymer chains. As a result, dimethacrylate-based resins exhibit greater mechanical strength and rigidity than PMMA [52].

Impact strength is an important intrinsic characteristic of denture base materials, representing their resistance to fracture after an accidental drop [54, 55]. It has been reported that 80% of mandibular denture fractures are caused by impact forces [56]. The results of this study showed no significant difference in impact strength between the tested materials, although 3D-printed materials demonstrated slight superiority. Some studies reported similar observations, with no significant difference between 3D-printed and heat-cured materials [57–59]. Others reported the opposite [23, 60], and the diversity in conclusions could be attributed to the factors previously explained for the flexural strength test, as many variables could lead to different conclusions, especially with 3D-printed materials.

During their use, denture base materials are subjected to humid environments [43]. The objective of the artificial aging procedure in this study was to replicate the conditions inside the mouth and evaluate its effect on the material characteristics. The aging process had a noticeable effect on the surface properties of the materials but not on the mechanical properties. Similarly, a significant decrease in HM was reported for the HC material, while a slight decrease was associated with the ND and FL. This progressive decline in HM of the HC compared to that of the 3D-printed materials reveals more plastic/elastic deterioration on its surface, which might be related to the filler content of the materials, as the HC had fewer fillers than did the other groups. It is challenging to make additional inferences from these findings, as the composition of the 3D-printed materials is not disclosed by the manufacturers, and for this reason, it would be desirable to determine their composition. The flexural and impact strengths decreased slightly but not significantly with the aging process. It is worth mentioning that the surface and mechanical properties are not correlated, and one cannot replace the other to characterize the material properties after aging [61].

3D-printed materials are considered promising alternatives to conventional materials, as supported by the results of this study. However, the results published in this field should be interpreted with care, as the diverse distribution of conclusions may result from different factors, such as differences in resin composition, printing orientation, and postprinting polymerization procedures, which can have detrimental effects on the objects produced. Using one type of heat-cured material can be considered a limitation of this study. Another limitation is that the specimen dimensions did not simulate a real-time denture base. Further studies can be conducted using the same materials in a denture configuration to obtain more reliable results.

3D-printed denture base materials have demonstrated potential as alternatives to conventional heat-cured materials due to their physical and mechanical properties as supported by the results of this study. However, it should be noted that the conclusions might be affected if other resin materials or 3D printing methods are used.

## 5. Conclusions

The study revealed several important findings. First, 3D-printed resin materials (NextDent and Formlab) displayed a slightly lower DC than their heat-cured counterparts. In addition, while the studied resin materials met the recommendations outlined in ISO 20795-1-2013 regarding sorption and solubility, they exhibited inferior performance compared to heat-cured materials, particularly in terms of water sorption, with a significant difference observed. Moreover, at baseline, the surface properties, including the Vickers and Martens' hardness and impact strength of the 3D-printed materials, were found to be comparable with those of conventional heat-cured materials, and the flexural strength of the 3D-printed material surpassed that of its counterpart. The surface properties of the heat-cured material and 3D-printed materials were affected by the aging process in artificial saliva over a period of three months. An impact on both the Vickers and Martens' hardness was observed for the heat-cured material, and an impact on only the Vickers hardness was observed for the 3D-printed materials. However, the effect on the flexural and impact strengths was minimal and did not show any statistical significance.

## Figures and Tables

**Table 1:** Tested materials and manufacturer information.

Code	Manufacturing technology	Composition (wt.%)	Manufacturer
ND	Additive manufacturing	(i) Ethoxylated bisphenol A dimethacrylate (≥75) (ii) 7,7,9-Trimethyl-4,13-dioxo-3,14-diazahexadecane-1,16-diyl bismethacrylate (10–20) (iii) 2-Hydroxyethyl methacrylate (5–10) (iv) Silicon dioxide (5–10) (v) Titanium dioxide (< 0.1)	NextDent

FL	Additive manufacturing	(i) Methacrylate monomer (40–60) (ii) Diurethane dimethacrylate (30–50) (iii) Propylidynetrimethyl trimethacrylate (5–10)	Formlabs

HC	Heat-cured manufacturing	Polymethyl methacrylate (>98)	Schottlander

**Table 2:** Experimental details for measuring.

Property	Sample size and group size	Equipment specification	Standards used	Equations	Measurement parameters
Vickers microhardness	*n* = 5	FM-700, Future-Tech Corp, Tokyo, Japan	—	*HV*=1.854*F*/*D*^2^ *F* is the applied load in *N* *D* is the mean diagonal length in mm	(i) Number of indentations: 3 (ii) After 24 hours postmanufacture (iii) After 3 months of aging in artificial saliva (iv) Load: 50 g

Martens hardness	*n* = 5 Diameter: 15.0 ± 0.2 mm, thickness: 2.0 ± 0.2 mm	Z2.5, ZwickRoell Ltd., Leominster, UK	ISO 14577-4/2016	HM=*F*/As (*h*)=*F*/26.43*∗h*^2^ in N/mm^2^ *F* represents the load in *N* *As*(*h*) is the surface area of the indenter at a distance of h from the tip, given in mm^2^	(i) Loading speed: 5 N/s (ii) Applied force: up to 50 N for 30 s (iii) Indenter tip's initial approach speed: 40 mm/min sensor tip position from specimen: 40 *μ*m (iv) Number of indentations: 5 (v) Vickers indentation tip: 136° (vi) Software: testXpert®, Zwick GmbH and Co, Ulm, Germany

Flexural modulus and strength	*n* = 10 Length: 64 ± 0.5 mm Width: 10.0 ± 0.2 mm Thickness: 3.3 ± 0.2 mm	Zwick/Roell Z020, Leominster, UK	ISO 20795-1: 2013	Σ=(3*Fl*/2*bh*^2^) in N/mm^2^ *E*=(*F*^1^*l*^3^/4*bh*^3^*d*) in N/mm^2^ *F* denotes the maximum applied force in N l represents the distance between the supports in mm *b* indicates the width of the specimen in mm *h* denotes the height of the specimen in mm *d* represents the deflection in mm at a load of F1 F1 denotes the load in Newton's at a position along the straight line of the load/deflection curve	(i) Load cell capacity: 500 N (ii) Horizontal distance between two supports: 50 ± 0.1 mm (iii) Crosshead preload speed: 5 mm/min

Impact strength	*n* = 10 Length: 80 ± 0.5 mm Width: 10 ± 0.2 mm Thickness: 4 ± 0.2 mm	Zwick/Roell Z020, Leominster, UK	EN ISO 179-1: 2010	acU = (*Wʙ*/*bh*) × 10³ in kJ/m^2^ *Wʙ* is the energy at break in joules *b* is the width of the specimen in mm, and *h* is the thickness of the specimen in mm	(i) Charpy unnotched impact test (ii) Horizontal distance between two supports: 40 ± 0.2 mm (iii) Freely swinging pendulum load cell: 4.0 J

**Table 3:** Filler content in the denture base materials.

Resin material	Filler content (wt.%) determined	Filler content (wt.%) supplied by manufacturer
HC	Not measurable	<2
FL	13.0 (2.2)^A^	Not available
ND	15.2 (2.8)^A^	6–15

^*∗*^In a column, cells with the same letters are not considered significantly different.

## Data Availability

The data used to support the findings of this study are included within the article.

## References

[1] Paulino M. R., Alves L. R., Gurgel B. C., Calderon P. S. (2015). Simplified versus traditional techniques for complete denture fabrication: a systematic review. *The Journal of Prosthetic Dentistry*.

[2] Al‐Dwairi Z. N., Tahboub K. Y., Baba N. Z., Goodacre C. J., Özcan M. (2019). A comparison of the surface properties of CAD/CAM and conventional polymethylmethacrylate (PMMA). *Journal of Prosthodontics*.

[3] Infante L., Yilmaz B., McGlumphy E., Finger I. (2014). Fabricating complete dentures with CAD/CAM technology. *The Journal of Prosthetic Dentistry*.

[4] Alp G., Murat S., Yilmaz B. (2019). Comparison of flexural strength of different CAD/CAM PMMA‐based polymers. *Journal of Prosthodontics: Official Journal of the American College of Prosthodontists*.

[5] Steinmassl O., Offermanns V., Stöckl W., Dumfahrt H., Grunert I., Steinmassl P.-A. (2018). In vitro analysis of the fracture resistance of CAD/CAM denture base resins. *Materials*.

[6] Bidra A. S., Taylor T. D., Agar J. R. (2013). Computer-aided technology for fabricating complete dentures: systematic review of historical background, current status, and future perspectives. *The Journal of Prosthetic Dentistry*.

[7] Bidra A. S., Farrell K., Burnham D., Dhingra A., Taylor T. D., Kuo C.-L. (2016). Prospective cohort pilot study of 2-visit CAD/CAM monolithic complete dentures and implant-retained overdentures: clinical and patient-centered outcomes. *The Journal of Prosthetic Dentistry*.

[8] Bilgin M. S., Baytaroglu E. N., Erdem A., Dilber E. (2016). A review of computer-aided design/computer-aided manufacture techniques for removable denture fabrication. *European Journal of Dermatology*.

[9] Kattadiyil M. T., AlHelal A. (2017). An update on computer-engineered complete dentures: a systematic review on clinical outcomes. *The Journal of Prosthetic Dentistry*.

[10] Shim J. S., Kim J. E., Jeong S. H., Choi Y. J., Ryu J. J. (2020). Printing accuracy, mechanical properties, surface characteristics, and microbial adhesion of 3D-printed resins with various printing orientations. *The Journal of Prosthetic Dentistry*.

[11] Lin C. T., Lee S. Y., Tsai T. Y., Dong D. R., Shih Y. H. (2000). Degradation of repaired denture base materials in simulated oral fluid. *Journal of Oral Rehabilitation*.

[12] Aati S., Akram Z., Shrestha B. (2022). Effect of post-curing light exposure time on the physico–mechanical properties and cytotoxicity of 3D-printed denture base material. *Dental Materials*.

[13] Zafar M. S. (2020). Prosthodontic applications of polymethyl methacrylate (PMMA): an update. *Polymers*.

[14] Gad M. M., Alshehri S. Z., Alhamid S. A. (2022). Water sorption, solubility, and translucency of 3D-printed denture base resins. *Dentistry Journal*.

[15] Perea-Lowery L., Gibreel M., Vallittu P. K., Lassila L. V. (2021). 3D-printed vs. heat-polymerizing and autopolymerizing denture base acrylic resins. *Materials*.

[16] Bartoloni J., Murchison D., Wofford D., Sarkar N. (2000). Degree of conversion in denture base materials for varied polymerization techniques 1. *Journal of Oral Rehabilitation*.

[17] Ferracane J., Hilton T., Stansbury J. (2017). Academy of Dental Materials guidance—resin composites: Part II—technique sensitivity (handling, polymerization, dimensional changes). *Dental Materials*.

[18] Broitman E. (2017). Indentation hardness measurements at macro-micro-and nanoscale: a critical overview. *Tribology Letters*.

[19] Liebermann A., Wimmer T., Schmidlin P. R. (2016). Physicomechanical characterization of polyetheretherketone and current esthetic dental CAD/CAM polymers after aging in different storage media. *The Journal of Prosthetic Dentistry*.

[20] Shahdad S. A., McCabe J. F., Bull S., Rusby S., Wassell R. W. (2007). Hardness measured with traditional Vickers and Martens hardness methods. *Dental Materials*.

[21] Hampe R., Lümkemann N., Sener B., Stawarczyk B. (2018). The effect of artificial aging on Martens hardness and indentation modulus of different dental CAD/CAM restorative materials. *Journal of the Mechanical Behavior of Biomedical Materials*.

[22] Altarazi A., Haider J., Alhotan A., Silikas N., Devlin H. (2023). 3D printed denture base material: the effect of incorporating TiO2 nanoparticles and artificial ageing on the physical and mechanical properties. *Dental Materials*.

[23] Gad M. M., Fouda S. M., Abualsaud R. (2022). Strength and surface properties of a 3D‐printed denture base polymer. *Journal of Prosthodontics*.

[24] Greil V., Mayinger F., Reymus M., Stawarczyk B. (2023). Water sorption, water solubility, degree of conversion, elastic indentation modulus, edge chipping resistance and flexural strength of 3D-printed denture base resins. *Journal of the Mechanical Behavior of Biomedical Materials*.

[25] Pfeiffer P., Rosenbauer E.-U. (2004). Residual methyl methacrylate monomer, water sorption, and water solubility of hypoallergenic denture base materials. *The Journal of Prosthetic Dentistry*.

[26] Arima T., Murata H., Hamad T. (1996). The effects of cross‐linking agents on the water sorption and solubility characteristics of denture base resin. *Journal of Oral Rehabilitation*.

[27] Ghavami-Lahiji M., Firouzmanesh M., Bagheri H., Jafarzadeh Kashi T. S., Razazpour F., Behroozibakhsh M. (2018). The effect of thermocycling on the degree of conversion and mechanical properties of a microhybrid dental resin composite. *Restorative dentistry and endodontics*.

[28] Imazato S., Tarumi H., Kato S., Ebi N., Ehara A., Ebisu S. (1999). Water sorption, degree of conversion, and hydrophobicity of resins containing Bis-GMA and TEGDMA. *Dental Materials Journal*.

[29] Ferracane J., Berge H., Condon J. (1998). In vitro aging of dental composites in water—effect of degree of conversion, filler volume, and filler/matrix coupling. *Journal of Biomedical Materials Research*.

[30] Doğan A., Bek B., Cevik N., Usanmaz A. (1995). The effect of preparation conditions of acrylic denture base materials on the level of residual monomer, mechanical properties and water absorption. *Journal of Dentistry*.

[31] Garcia L. d. F. R., Roselino L. d. M. R., Mundim F. M., Pires‐de‐Souza F. d. C. P., Consani S. (2010). Influence of artificial accelerated aging on dimensional stability of acrylic resins submitted to different storage protocols. *Journal of Prosthodontics*.

[32] Wong D. M., Cheng L. Y., Chow T., Clark R. K. (1999). Effect of processing method on the dimensional accuracy and water sorption of acrylic resin dentures. *The Journal of Prosthetic Dentistry*.

[33] Asmussen E., Peutzfeldt A. (1998). Influence of UEDMA, BisGMA and TEGDMA on selected mechanical properties of experimental resin composites. *Dental Materials*.

[34] Ilie N., Hickel R. (2009). Investigations on mechanical behaviour of dental composites. *Clinical Oral Investigations*.

[35] Kim K.-H., Ong J. L., Okuno O. (2002). The effect of filler loading and morphology on the mechanical properties of contemporary composites. *The Journal of Prosthetic Dentistry*.

[36] Altarazi A., Haider J., Alhotan A., Silikas N., Devlin H. (2022). Assessing the physical and mechanical properties of 3D printed acrylic material for denture base application. *Dental Materials*.

[37] Cucci A. L. M., Vergani C. E., Giampaolo E. T., Afonso M. C. S. F. (1998). Water sorption, solubility, and bond strength of two autopolymerizing acrylic resins and one heat-polymerizing acrylic resin. *The Journal of Prosthetic Dentistry*.

[38] Berli C., Thieringer F. M., Sharma N. (2020). Comparing the mechanical properties of pressed, milled, and 3D-printed resins for occlusal devices. *The Journal of Prosthetic Dentistry*.

[39] Kawaguchi T., Lassila L. V., Sasaki H., Takahashi Y., Vallittu P. K. (2014). Effect of heat treatment of polymethyl methacrylate powder on mechanical properties of denture base resin. *Journal of the Mechanical Behavior of Biomedical Materials*.

[40] Al-Harbi F. A., Abdel-Halim M. S., Gad M. M. (2019). Effect of nanodiamond addition on flexural strength, impact strength, and surface roughness of PMMA denture base. *Journal of Prosthodontics: Official Journal of the American College of Prosthodontists*.

[41] Prpic V., Schauperl Z., Catic A., Dulcic N., Cimic S. (2020). Comparison of mechanical properties of 3D-printed, CAD/CAM, and conventional denture base materials. *Journal of Prosthodontics*.

[42] Polychronakis N., Dimitriadi M., Ioannidis A., Papadopoulos T. (2020). The effect of different cooling procedures on mechanical properties of denture base materials measured by instrumented indentation testing. *Journal of Prosthodontic Research*.

[43] Reymus M., Stawarczyk B. (2021). In vitro study on the influence of postpolymerization and aging on the Martens parameters of 3D-printed occlusal devices. *The Journal of Prosthetic Dentistry*.

[44] Fiore A. D., Meneghello R., Brun P. (2022). Comparison of the flexural and surface properties of milled, 3D-printed, and heat polymerized PMMA resins for denture bases: an in vitro study. *Journal of Prosthodontic Research*.

[45] Puebla K., Arcaute K., Quintana R., Wicker R. B. (2012). Effects of environmental conditions, aging, and build orientations on the mechanical properties of ASTM type I specimens manufactured via stereolithography. *Rapid Prototyping Journal*.

[46] Zhang Z.-c., Li P.-l., Chu F.-t., Shen G. (2019). Influence of the three-dimensional printing technique and printing layer thickness on model accuracy. *Journal of Orofacial Orthopedics/Fortschritte der Kieferorthopädie*.

[47] Dimitrov D., Schreve K., de Beer N. (2006). Advances in three dimensional printing–state of the art and future perspectives. *Rapid Prototyping Journal*.

[48] Vayrynen V. O. E., Tanner J., Vallittu P. K. (2016). The anisotropicity of the flexural properties of an occlusal device material processed by stereolithography. *The Journal of Prosthetic Dentistry*.

[49] Unkovskiy A., Bui P. H. B., Schille C., Geis-Gerstorfer J., Huettig F., Spintzyk S. (2018). Objects build orientation, positioning, and curing influence dimensional accuracy and flexural properties of stereolithographically printed resin. *Dental Materials*.

[50] Rodrigues S. A., Scherrer S. S., Ferracane J. L., Bona Á. D. (2008). Microstructural characterization and fracture behavior of a microhybrid and a nanofill composite. *Dental Materials*.

[51] Ferracane J. L. (1995). Current trends in dental composites. *Critical Reviews in Oral Biology and Medicine*.

[52] Anusavice K. J., Shen C., Rawls H. R. P. (2012). *Science of Dental Materials*.

[53] Ratner B. D., Hoffman A. S., Schoen F. J., Lemons J. E. (2004). *Biomaterials Science: An Introduction to Materials in Medicine*.

[54] Faot F., Costa M. A., Del Bel Cury A. A., Rodrigues Garcia R. C. (2006). Impact strength and fracture morphology of denture acrylic resins. *The Journal of Prosthetic Dentistry*.

[55] da Cruz Perez L. E., Lucia Machado A., Eduardo Vergani C., Andrade Zamperini C., Cláudia Pavarina A., Vicente Canevarolo S. (2014). Resistance to impact of cross-linked denture base biopolymer materials: effect of relining, glass flakes reinforcement and cyclic loading. *Journal of the Mechanical Behavior of Biomedical Materials*.

[56] Sasaki H., Hamanaka I., Takahashi Y., Kawaguchi T. (2016). Effect of long-term water immersion or thermal shock on mechanical properties of high-impact acrylic denture base resins. *Dental Materials Journal*.

[57] Al‐Dwairi Z. N., Al Haj Ebrahim A. A., Baba N. Z. (2023). A comparison of the surface and mechanical properties of 3D printable denture‐base resin material and conventional polymethylmethacrylate (PMMA). *Journal of Prosthodontics*.

[58] Lee J. (2020). *Impact Strength of 3D Printed and Conventional Heat-Cured and Cold-Cured Denture Base Acrylics*.

[59] Alshaikh A. A., Khattar A., Almindil I. A. (2022). 3D-Printed nanocomposite denture-base resins: effect of ZrO2 nanoparticles on the mechanical and surface properties in vitro. *Nanomaterials*.

[60] Chhabra M., Nanditha Kumar M., RaghavendraSwamy K., Thippeswamy H. (2022). Flexural strength and impact strength of heat-cured acrylic and 3D printed denture base resins-A comparative in vitro study. *Journal of Oral Biology and Craniofacial Research*.

[61] Fischer J., Roeske S., Stawarczyk B., Hämmerle C. H. F. (2010). Investigations in the correlation between Martens hardness and flexural strength of composite resin restorative materials. *Dental Materials Journal*.

